# Environmental factors effecting the germination and seedling emergence of two populations of an aggressive agricultural weed; *Nassella trichotoma*

**DOI:** 10.1371/journal.pone.0199491

**Published:** 2018-07-05

**Authors:** Talia Humphries, Bhagirath S. Chauhan, Singarayer K. Florentine

**Affiliations:** 1 Centre for Environmental Management, Faculty of Science and Technology, Federation University Australia, Mount Helen, Victoria, Australia; 2 Centre for Plant Science, Queensland Alliance for Agriculture and Food Innovation (QAAFI), The University of Queensland, Toowoomba, Queensland, Australia; College of Agricultural Sciences, UNITED STATES

## Abstract

*Nassella trichotoma* (Nees) Hack. ex Arechav. (Serrated tussock) is an aggressive globally significant weed to agricultural and natural ecosystems. Herbicide resistant populations of this C_3_ perennial weed have emerged, increasing the need for effective wide-scale cultural control strategies. A thorough seed ecology study on two spatially distinct populations of *N*. *trichotoma* was conducted on this weed to identify differences in important environmental factors (drought, salinity, alternating temperature, photoperiod, burial depth, soil pH, artificial seed aging, and radiant heat) which influence seed dormancy. Seeds were collected from two spatially distinct populations; Gnarwarre (38 ^O^ 9' 8.892'' S, 144 ^O^ 7' 38.784'' E) and Ingliston (37^O^ 40' 4.44'' S, 144 ^O^ 18' 39.24'' E) in December 2016 and February 2017, respectively. Twenty sterilized seeds were placed into Petri dishes lined with a single Whatman® No. 10 filter paper dampened with the relevant treatments solution and then incubated under the identified optimal alternating temperature and photoperiod regime of 25°C/15°C (light/dark, 12h/12h). For the burial depth treatment, 20 seeds were placed into plastic containers (10cm in diameter and 6cm in depth) and buried to the relevant depth in sterilized soil. All trials were monitored for 30 days and germination was indicated by 5mm exposure of the radicle and emergence was indicated by the exposure of the cotyledon. Each treatment had three replicates for each population, and each treatment was repeated to give a total of six replicates per treatment, per population. *Nassella trichotoma* was identified to be non-photoblastic, with germination (%) being similar under alternating light and dark and complete darkness conditions. With an increase of osmotic potential and salinity, a significant decline in germination was observed. There was no effect of pH on germination. Exposure to a radiant heat of 120°C for 9 minutes resulted in the lowest germination in the Ingliston population (33%) and the Gnarwarre population (60%). In the burial depth treatment, the Ingliston population and the Gnarwarre population had highest emergence of 75% and 80%, respectively at a depth of 1cm. Variation between the two populations was observed for the burial depth treatments; Gnarwarre had greater emergence than Ingliston from the 4cm burial depth, while Ingliston had greater emergence at the soil surface than Gnarwarre. The Gnarwarre population had greater overall germination than Ingliston, which could be attributed to the greater seed mass (0.86mg compared to 0.76mg, respectively). This study identifies that spatial variations in *N*. *trichotoma’s* seed ecology are present between spatially distinct populations.

## Introduction

The ability for aggressive weeds to germinate and emerge vigorously allows them to dominate and displace desirable species. Therefore, an understanding of seed ecology is essential for developing effective management programs for problematic weed species. Weeds are usually most susceptible to control methods, making control strategies targeted to early life stages highly effective [1, 2]. Abiotic factors such as drought, light, salinity, seed burial depth, soil pH, and temperature as well as disturbance events such as a fire, flooding or tillage can play an important role in initiating or inhibiting seed germination [3–4]. Therefore, in order to develop smarter and more effective control strategies for aggressive weeds like *Nassella trichotoma* (Nees) Hack. ex Arechav., a comprehensive study into the requirements for their successful germination, seedling emergence and subsequent establishment should be investigated [5, 6, 7]. By identifying the parameters which positively or negatively influence seed germination and seedling vigour, suitable management strategies can be developed to reduce the successful establishment of seedlings and deplete the soil stored seedbank [8].

High reproductive output is a key trait of successful weeds. A high density of seeds in the soil seedbank can give a weed a competitive advantage over crops or native plant species, particularly if the weed species is faster growing than desirable species. Dense seedbanks can cause persistent management challenges. *Nassella trichotoma* of the Poaceae family is problematic weed can produce over 140,000 seeds per plant on an annual basis, allowing it to quickly dominate the soil seedbank [9–10]. It has been identified that between 74% to 91% of *N*. *trichotoma* seeds will germinate within their first six to twelve months, with some seeds remaining dormant in the soil for up to three years before losing their viability [11]. Dormancy is an internal feature of a seed that prevents germination, even when environmental conditions are adequate [1, 12]. Once a seed has initiated the germination process it, it cannot be stopped. Therefore, to ensure the best chance of successful growth and survival, dormancy break is strongly linked with specific environmental cues. Understanding dormancy patterns for invasive weeds has important implications for their management [12, 13]. Many weed species, including *N*. *trichotoma* undergo a brief period of non-deep physiological dormancy [4]. This type of dormancy is strongly associated with seasonality, particularly alternating temperatures. Non-deep physiological dormancy is caused by a physiological mechanism within the seeds embryo that requires specific stimulation, before the radical will emerge [5]. This trait allows *N*. *trichotoma* to avoid germinating in summer after seed drop and allows it to wait for more suitable wetter and cooler conditions [14].

Studies have shown that light and alternating temperature regimes have been identified as two of the most important environmental factors in triggering seed germination [4, 15, 16]. Photochromes within an imbibed seed allow identification of the intensity of competition within its environment [17–18]. The ability to detect competition prior to germination may improve seedling survival rates [17]. Ratios of far-red to red light are higher in environments with intense competition, as the more favourable red light is absorbed by established plants, therefore less red light reaches the soil surface [17]. In an environment where competition is low, red light will be detected in higher ratios than far-red light by the seed, promoting the germination process [19]. Seeds which germinate under completely dark conditions may have an abundance of far-red phytochromes within their embryonic tissue, helping them to identify intense competition [12, 20]. Researchers have identified that light can promote significantly higher germination in many plants species including *Halocnemum strobilaceum* [21], *Leptochloa chinensis* [22], *Carduus nutans* [23], and *Echinochloa colona* [5]. By identifying if a weed is positive photoblastic, light restrictive management strategies such as mulching using crop residue [24–25] or developing dense perennial competition [26] can be introduced for effective control. Despite the implications of light sensitivity on successful recruitment, there are also many plants that exhibit light independent germination [4, 6]. Light independent germination is closely linked to other environmental triggers, particularly temperature [4, 19]. Temperature breaks dormancy by altering seed physiology and has been observed to influence the rate and percentage of germination, although this effect varies greatly by species [4, 15]. For example, optimal alternating temperature regimes were found to break the dormancy of, and hence significantly increase germination in *Moehringia trinervia* seeds, in contrast to this, it had minimal effect on *Stellaria nemorum* [27]. Therefore, while temperature is an important trigger for breaking seed dormancy in some species, like *M*. *trinervia*, different environmental factors such as rainfall and soil type can also play an important role in triggering the germination process.

Seeds buried deeper into the soil profile often have lower success rates of emergence and establishment due to the amount of energy required to reach the soil surface. The size of a seed may determine the depth from which it emerges; large seeds may have greater energy reserves, allowing emergence from greater depths than smaller seeds. The effect of seed size on emergence was observed in four species of Amaranthus, with the lightest species (*Amaranthus* spinosus) having significantly shallower optimal burial depth compared to the denser species (*Amaranthus* viridis) [28]. Germination of photoblastic seeds decrease with an increase in burial depth. Seed burial has been observed to significantly reduce seedling emergence in light dependent weeds such *Eclipta prostrata* [29], *L*. *chinensis* [22], and *Murdannia nudiflora* [24]. Depending on the vigour of the seed, those species that germinate independent of light can also be restricted by increased burial, as observed in the desert weed *Marrubium vulgare* [30]. By identifying the burial depth from which weed seedlings cannot emerge, recommendations in tillage depths for control can be proposed.

Seed germination can be linked to other environmental factors. Low moisture availability can prolong dormancy as soil moisture levels may be insufficient for imbibition and competitive emergence of seedlings [31]. This may prove problematic for *N*. *trichotoma* as mass germination events have been strongly linked to periods of heavy rainfall [14, 32]. In saline environments, the salt ions in the soil can reverse the natural osmotic flow of moisture into the dry seed and rather force water out of the seed, preventing imbibition. Increased salinity has been observed to significantly reduce seed germination in many weed species including *Cardaria draba* [33] and *Eragrostis plana* [31]. However, it generally does not affect their viability when these seeds were alleviated from the salinity stress, and normal germination was observed. It is common for weeds to tolerate a wide range of soil pH levels [4, 28, 33, 34] which is a key trait of an invasive generalist species. However, by identifying if particular soil factors, such as pH and salinity enhance germination, regions at risk will be easier to identify.

Fire can also play an important role in breaking seed dormancy and triggering germination events. Fire can remove established competition, allowing for greater light penetration to the soil surface, which can trigger germination in light sensitive seeds. The reduced competition allows for higher nutrient and moisture availability for seedling establishment. As weeds are generally good, fast-growing coloniser species, they can have a competitive advantage over desirable species. Due to fire being an important ecological management tool, it is important to understand how weeds like *N*. *trichotoma* respond to burn temperatures and durations. Fire may be a useful tool to decrease the seedbank if seed viability or establishment can be reduced [35]. On the contrary, fire can also act as a germination trigger, as observed for *N*. *trichotoma*, and utilized to promote a flush of seed germination from the seedbank before herbicide application [10, 32, 33, 36, 37].

Understanding how these environmental cues influence the germination of weeds may not be sufficient alone for developing wide scale strategic management plans. Weeds are considered to be pioneer species, which contributes to their wide dispersal and fast adaptability to a variety of ecosystems. The selective pressures exerted by these different ecosystems can, overtime, lead to in local adaptions between geographically distinct populations [38, 39]. This can result in one species responding differently to the same environmental cues based on the selective pressures acting on the population. Germination rates varied significantly between two spatially distinct populations of *Poa annua* in response to photoperiod, temperature and the fungicide, fenarimol [40]. Differences in temperature tolerance were observed in different populations of the widespread crop weeds *Galinsoga quadriradiata* and *G*. *parviflora*, with optimal germination under controlled conditions reflecting that of the given populations habitat [8].

*Nassella trichotoma* has adapted to a range of managed and natural ecosystems across the world, which may have resulted in spatially distinct populations exhibiting some variability in their seed ecology [41]. Phenotypic variations have been observed in the size and height of Australian populations with Victorian populations being notably smaller than those in New South Wales and Tasmanian [14]. In Victoria, some populations have been identified to exhibit resistance to flupropanate herbicide, requiring four times the recommended dose, which can be harmful to native plants and therefore reducing competition [42, 43]. By identifying any local adaptions, more specialised, ecosystem-specific management practices can be developed [38].

The objective of this study was to identify how the environmental factors of light, temperature, heat, salinity, drought, soil pH, and seed burial influence germination and seedling emergence of two *N*. *trichotoma* populations.

## Methods

### Seed collection and storage

Mature *N*. *trichotoma* seeds were collected from over 100 plants from two populations in Victoria, Australia; Ingliston (37^O^ 40' 4.44'' S, 144^O^ 18' 39.24'' E) and Gnarwarre (38 ^O^ 9' 8.892'' S, 144 ^O^ 7' 38.784'' E) during February 2017 and December 2016, respectively. Seeds were collected on private properties, and both landholders gave us a permission to collect seeds. Given that is a weed species and seeds were used for research purpose no further permission or approval required. These two populations are separated by approximately 100 km. The seeds were placed in labelled plastic, zip-lock bags and transported to Federation University Australia’s seed ecology lab. Seeds were stored within labelled plastic zip-lock bags at room temperature until the trials began in March 2017.

### Site description

**Fig 1:**
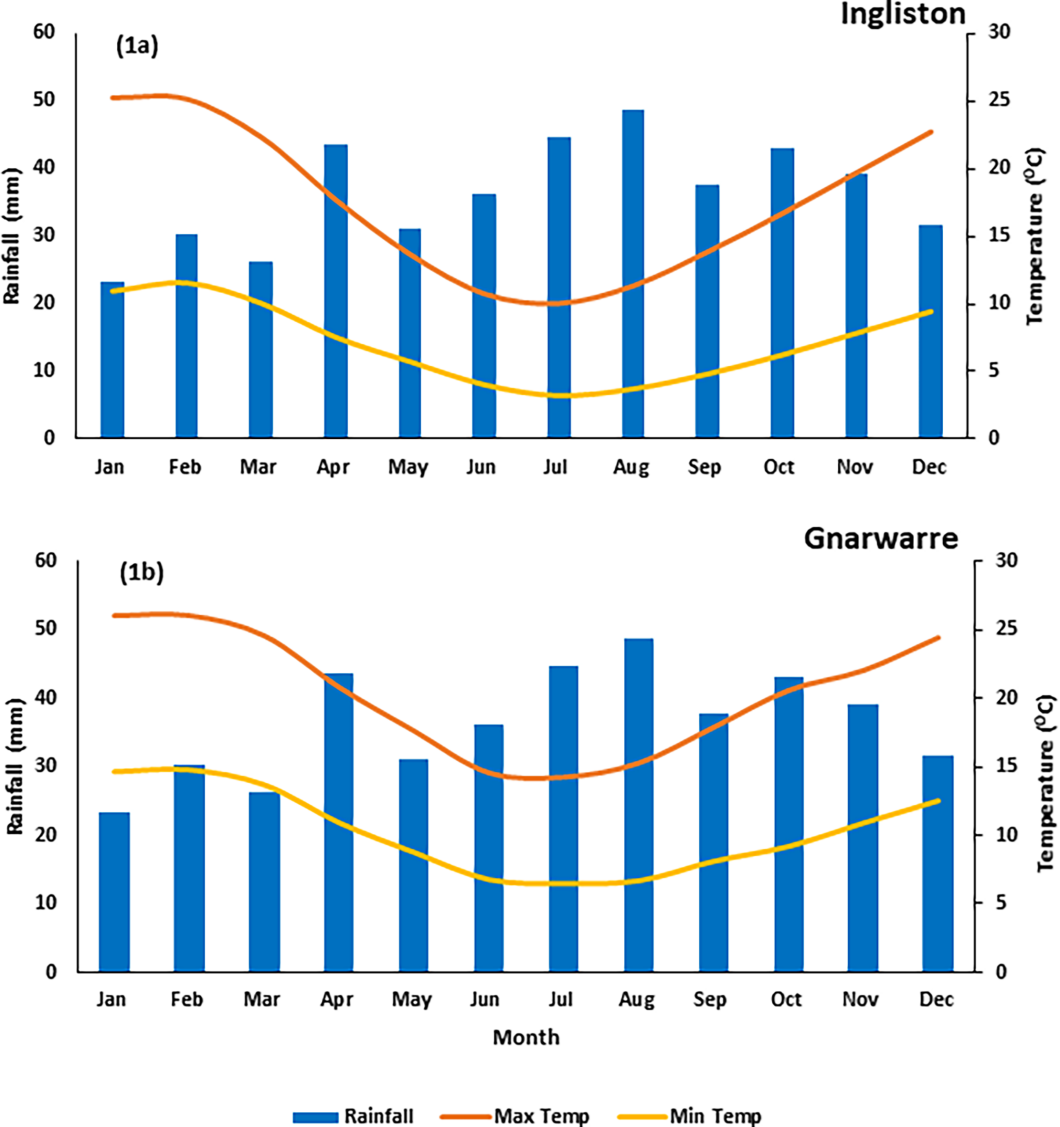
**a: The average monthly rainfall and maximum and minimum temperature collected from the closest weather stations with relevant and recent data to the Ingliston site.** The rainfall data was collected from the Pykes Creek station (37°36'40"S, 144°18'0"E) located approximately 10 km from the Ingliston site, and the data was averaged from November 1956 to August 2017, and the temperature data was collected from the Ballarat Aerodrome station (37°30'46"S, 143°47'28"E) located approximately 50 km from the Ingliston site, with the data averaged from January 1908 to August 2017. The information was sourced from the Bureau of Meteorology [49]. **b:** The average monthly rainfall and maximum and minimum temperature collected from the closest weather stations with relevant and recent data to the Gnarwarre site. The rainfall data was collected from the Gnarwarre station (38°8'37"S, 144°11'14"E) located approximately 5 km from the Gnarwarre site, and the data was averaged from October 1996 to August 2017, and the temperature data was collected from the Geelong Racecourse station (38°10'25"S, 144°22'35"E) located approximately 25 km from the Gnarwarre site, with the data averaged from June 2011 to August 2017. The information was sourced from the Bureau of Meteorology [49].

The Ingliston site is located on a privately owned eucalypt bushland within a valley, which offers the vegetation some protection from harsh winds. Aside from the established trees, this site was heavily infested with almost a monoculture of *N*. *trichotoma*. A soil analysis was conducted to identify pH and salinity. The soil pH of 4.5 was identified by using a Manutec soil pH test kit. The soil salinity was measured using the 1:5 soil:water ratio methods described by Slavich & Patterson [44], and was identified to be 3.8dS/m, which is considered to be slightly saline [45]. Ingliston receives its highest rainfall throughout August (49ml), and the average temperature ranges from a maximum of 25°C in summer to a minimum of 3°C during the winter (Fig 1A) [46].

The Gnarwarre site is located on a privately owned pastoral field for grazing sheep. The site is located on an open hill, with little shelter from the elements. Pastural grasses provide intense competition for this population of *N*. *trichotoma*, resulting in the population size being smaller than that at Ingliston. The same soil analysis techniques used for the Ingliston site were applied to Gnarwarre, and identified the soil to have a pH of 6 and soil salinity of 4.3dS/m, which is considered to be moderately saline [45]. Gnarwarre receives its highest rainfall throughout August (49ml), and the average temperature ranges from 26°C in summer to 6°C during the winter (Fig 1B) [46].

### Seed preparation

Seeds were assumed to be viable when they had a plump appearance and a soft “clink” was heard when the seed was dropped into the petri dish. All the trials had three replicates with 20 randomly selected seeds in each, which were repeated to give a total of six replicates (120 seeds) per treatment. All seeds were sterilized using 1% sodium hypochlorite for 5 minutes and then were thoroughly rinsed with sterilized reverse osmosis (RO) water. All trials had 20 sterilized seeds placed into each plastic Petri dish lined with a single layer of sterilized Whatman® No. 10 filter paper and then moistened with 10ml of the relevant solution. The Petri dishes were wrapped with a strip of parafilm to maintain moisture, and germination was counted weekly for 30 days. Germination was determined when approximately 2mm of the radicle was visible and the cotyledon had emerged from the seed coat [47]. At the conclusion of the treatments, any un-germinated seeds were tested for their viability using 2,3,5-triphenyltetrazolium chloride (TTC) test [48, 49].

### The effect of photoperiod and alternating temperature

Determination of the photoperiod and temperature range that generates the highest germination percentage for *N*. *trichotoma* is essential for the success of all subsequent experiments. Replicates were placed into one of four incubation cabinets (Thermoline Scientific and Humidity Cabinet, TRISLH-495-1-SD, Vol. 240, Australia) fitted with cool-white fluorescent lamps that provided a photosynthetic photon flux of 40μmol m^-2^ s^-1^ set at various temperature regimes: 17/7, 25/15, 30/20 and 40/30°C, each alternating 12 hours light and 12 hours dark. To prevent excessive water loss, the dishes exposed to the light and dark treatments had a strip of parafilm wrapped around the outside of each Petri dish, and the 24-hour dark replicates were covered in a double layer of aluminum foil, which also blocked out light. To ensure appropriate conditions for the 24-hour dark treatment, seeds were not subjected to any white light, which was assured by the practice of examining Petri dishes containing these seeds under a green safe light. The dishes exposed to the 40/30°C treatments also had an additional strip of cling wrap placed over the parafilm as an added precaution, as the parafilm was observed to melt at this temperature regime.

### The effect of drought

To identify the effects of drought on germination, polyethylene glycol 8000® (PEG, Sigma-Aldrich Co., 3050, Spruce St., MO 63103 in sterilized distilled water) was dissolved into sterilized RO water to make aqueous osmotic potential solutions of 0 (sterilized RO water for the control), -0.1, -0.2, -0.4, -0.6, -0.8, and -1.0MPa. To make 500ml of each solutions for the average temperature of 20°C, PEG was weighed out using an electric scale and added to a flask containing 500ml of RO water and stirred automatically until dissolved. The solutions were placed into a labelled bottle wrapped in aluminium foil and stored in a fridge until use. The concentrations used for each solution was 46.8, 66.175, 93.575, 114.6, 132.35, and 147.95g to make the -0.1, -0.2, -0.4, -0.6, -0.8, and -1MPa solutions, respectively. The filter papers were dampened with 10ml of the relevant solution, and the dishes were incubated under alternating temperatures of 25/15°C, 12 hours light and 12 hours dark.

### The effect of salinity

The effect of salinity on *N*. *trichotoma* germination was examined by using sodium chloride (NaCl) solutions of 0 (sterilized RO water for the control), 25, 50, 100, 150, 200, and 250mM. This range of NaCl concentrations was selected to reflect the level of salinity occurring in typical Australian disturbed soil [50]. Approximately 10ml of the relevant saline solution was used to dampen the filter paper, and the petri dishes were incubated under alternating temperatures of 25/15°C, 12 hours light and 12 hours dark.

### The effect of seed burial

To test the impact of seed burial on germination and subsequent seedling emergence, the seeds were placed at depths of 0 (surface), 1, 2, 3 and 4cm in sterilized soil. Soil was collected from the Ingliston site (37^O^ 40' 4.44'' S, 144^O^ 18' 39.24'' E) and sterilized in an autoclave at Federation University (Victoria), to kill other seeds and propagules. Soil was sieved using a 2cm sieve and stored in a sealed 100L plastic tub until use. Round plastic containers 10cm in diameter and 6cm in depth were prepared by drilling small holes into the bottom of each to allow the percolation of water into the soil. Each container had a single layer of cleaning cloth placed at the base prior to being filled with soil and burying the seeds. The trials were placed into large white trays (28cm x 44cm x 5.5cm), which were lined with two sheets of cleaning cloth. The trays were initially filled with 500ml of RO water, and this amount was added on every alternating day. The trials were housed in the incubation cabinets under alternating temperatures of 25/15°C, 12 hours light and 12 hours dark. Seedling emergence was monitored on alternating days. Emergence was indicated by the cotyledon protruding from the soils surface.

### Seed longevity under field conditions

In order to determine the effect of burial depth on seed viability under field conditions, 120 viable seeds from the Ingliston population were randomly selected and placed into a 5cm X 5cm semi-permeable bag made of 0.5mm aluminium mesh that allowed for the natural flow of water and micro pathogens, while keeping the seeds contained. A total of 24 bags containing 120 seeds each were made in total and they were sealed using a hot glue gun. The mesh bags were then buried at a randomly selected site within the location of seed collection at Ingliston, Victoria (37^O^ 40' 4.44'' S, 144 ^O^ 18' 39.24'' E). The bags were buried at depths of 0 (surface), 1, 2 and 4cm. One bag from each depth was collected each month and returned to Federation University Australia, seed ecology lab where germinated seeds were counted and removed from the mesh bag. The remaining seeds had excess dirt removed using tap water and up to 20 seeds were plated into Petri dishes lined with a single layer of sterilized Whatman® No. 10 filter paper and then moistened with 10ml of sterilized RO water. The Petri dishes were placed into an incubation cabinet at alternating temperatures of 25/15°C, 12 hours light and 12 hours dark.

### The effect of heat shock

The effect of heat on seed germination and viability was examined by exposing the seeds to five temperatures; 40, 60, 80, 100, and 120°C. Furthermore, at each temperature, seeds were exposed to the heat for three durations; 3, 6 or 9 minutes. Seeds were placed circular into aluminum trays (8cm diameter and 3cm depth) and then placed into a digital oven (Memmert, Type No. ULE500) at the relevant temperature for the required duration. Once removed, they were immediately plated on plastic Petri dish lined with a single layer of sterilized Whatman® No. 10 filter paper and then moistened with sterilized RO water and placed into an incubation cabinet under alternating temperatures of 25/15°C, 12 hours light and 12 hours dark.

### The effect of pH

The effect of pH on seed germination was determined by dampening the filter papers with relevant buffer solutions ranging from pH 4 through to pH 10, prepared according to the method described by Chachalis and Reddy [51]. Potassium hydrogen phthalate was adjusted to pH 4 using 1 N of hydrogen chloride (HCl). The buffer solutions of pH 5 and 6 were prepared by altering 2mM of MES [2-(N-morpholino) ethanesulfonic acid] with 1 N of sodium hydroxide (NaOH). To make the buffer solutions of pH 7 and 8, 2mM of HEPES [N-(2-hydroxymethyl) piperazine–N–(2- ethane sulfonic acid)] was adjusted using 1 N of NaOH. The buffer solutions of pH 9 and 10 were created by adjusting a 2mM solution of Tricine [N-Tris (hydroxymethyl) methyl glycine] with 1 N of NaOH. The dishes were incubated at an alternating light and temperature regime of 25/15°C, 12 hours light and 12 hours dark.

### Statistical analyses

The final germination percentage (FG%) was calculated dividing the sum of germinated seeds (SG) by the total number (TS) of seeds placed into each Petri dish:

$$FG=\frac{SG}{TS}\times 100$$

The average germination percentage (G%) and standard error was calculated for each treatment, and these values for all the treatments except for the rate of germination data, were entered into the statistical software SigmaPlot 13 (Systat Software, Inc., Point Richmond, CA, USA) for analysis. The rate of germination was analysed using Microsoft Excel. The effect of drought on germination percentage was fitted with a polynomial linear model:

G%=y0+a×x

-where, *G*% is the averaged germination (%) at the osmotic potential of *x* and *a* indicates the slope.

The effect of salinity on germination percentage was fitted with a three-parameter sigmoid model:

$$G\%=a/(1+e\left(-\frac{x-x0}{b}\right))$$

-where, *G*% is the total germination (%) at the NaCl concentration of *x* and *b* indicates the slope, *a* is the maximum emergence (%) and *x*0 is defined as the concentration for 50% inhibition of the maximum germination (%) as a result of the treatment.

The effect of burial depth on seedling emergence was fitted with a three-parameter peak Gaussian model:

$$E\%=a\times e(-0.5\times \frac{\left(x-x0\right)}{b}{)}^{2}$$

-where, *E*% indicates the emergence (%), *a* is the maximum emergence (%), *b* indicates the slope, and *x*0 is defined as the concentration for 50% inhibition of the maximum germination (%) as a result of the treatment.

A two-way ANOVA was generated for each treatment by using a general linear model on with the statistical program Minitab.

## Results and discussion

### The effect of photoperiod and alternating temperature on germination (%)

**Fig 2:**
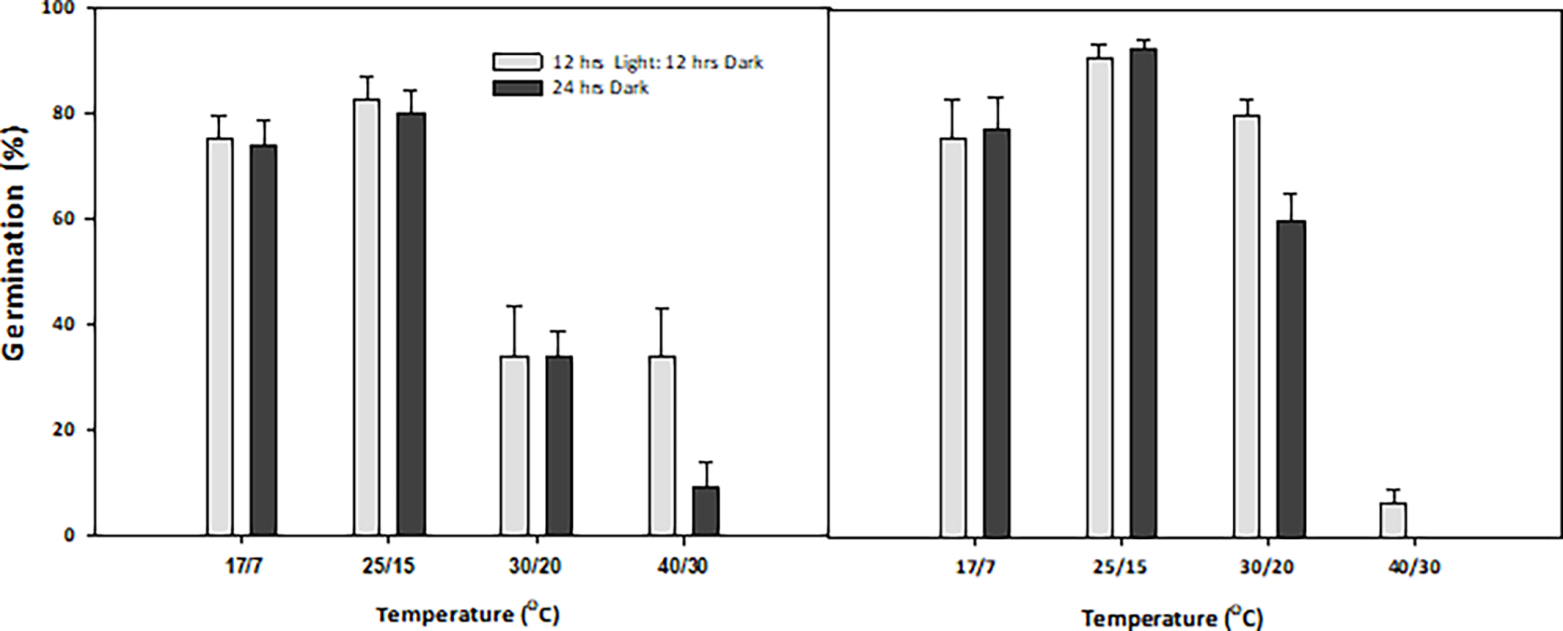
**a: The effect of alternating temperature and photoperiod regimes on the germination (%) of *Nassella trichotoma* seeds for the Ingliston and b: Gnarwarre populations after incubation in a growth chamber for 30 days.** Vertical bars represent standard error of the mean.

Both *N*. *trichotoma* populations had the highest germination (%) at the alternating temperature of 25/15°C (Fig 2A and 2B). Under the alternating photoperiod of 12 hours light and 12 hours dark at this temperature, the Ingliston population had 82.5% germination and Gnarwarre had 90.8%, and similar counts were obtained in complete darkness with 80% and 92.5% germination for the Ingliston and Gnarwarre populations, respectively. Both populations also demonstrated high germination (%) under the alternating temperature of 17/7°C under both alternating light and dark (75% and 75.8% for Ingliston and Gnarwarre, respectively) and complete darkness photoperiods (74.16% and 77.5% for Ingliston and Gnarwarre, respectively). For both populations, germination (%) was significantly reduced under the alternating temperature of 40/30°C (p = 0.000), with a total germination (%) for the Ingliston population of 34.2% (alternating) and 9.2% (complete darkness), and was even further reduced for the Gnarwarre population, which had a germination (%) of only 6.7% (alternating) and 0.8% (complete darkness). At the alternating temperature of 30/20°C, the 12 hours light and 12 hours dark photoperiod significantly enhanced the germination (%) in the Gnarwarre population (p = 0.002), with a total germination of 80.8% for the alternating photoperiod and only 60.8% under complete darkness. At this temperature, germination (%) was significantly reduced compared to the 17/7°C and 25/15°C treatments within the Ingliston population, having a total germination (%) of only 34.1% for both photoperiods. The germination (%) for Ingliston in the 30/20°C alternating photoperiod treatment was also lower compared to the Gnarwarre population at the same light and temperature regime (p = 0.028). Temperature was a more influential factor on germination (%) than photoperiod (Table 1). The high R-squared value of 89% shows that the results obtained are strongly associated with the treatment (Table 1).

**Table 1:** Statistical output of the two-way ANOVA created using a general linear model. The tested environmental parameters and their interactions are displayed. Statistically significant points are italicized in red. The r-squared value indicates the proportion of the result that is influenced directly by the treatment.

Treatment	Factors	Df	F-Value	Sig	R-Sq
Temperature and Photoperiod	Temperature	3	192.91	0.000	89.5%
	Population	1	10.63	0.002	
	Photoperiod	1	10.63	0.002	
	Temperature X Population	3	21.18	0.000	
	Temperature X Photoperiod	3	1.57	0.204	
	Population X Photoperiod	1	0.21	0.608	
	Temperature X Population X Photoperiod	3	3.19	0.028	
pH	pH Level	6	0.83	0.548	36.6%
	Population	1	27.31	0.000	
	pH X Population	6	1.36	0.244	
NaCl	NaCl Concentration	6	96.06	0.000	89.6%
	Population	1	11.61	0.001	
	NaCl X Population	6	2.49	0.031	
Heat	Temperature	4	22.21	0.000	70.2%
	Duration of Exposure	2	1.70	0.185	
	Population	1	229.31	0.000	
	Temperature X Duration of Exposure	8	1.74	0.093	
	Temperature X Population	4	1.84	0.129	
	Duration of Exposure X Population	2	0.03	0.966	
	Temperature X Duration of Exposure X Population	8	1.24	0.280	
Burial Depth	Depth	4	9.18	0.000	49.0%
	Population	1	0.74	0.393	
	Depth X Population	4	2.63	0.045	
Drought	PEG Concentration	6	330.34	0.000	96.1%
	Population	1	50.46	0.000	
	PEG Concentration X Population	6	11.59	0.000	
Germination under field conditions	Depth	3	63.14	0.000	90.5%
	Month	5	0.22	0.951	
Artificial aging	Depth	3	15.79	0.000	70.3%
	Month	5	0.24	0.939	

### The effect of photoperiod and alternating temperature on rate of germination

**Fig 3:**
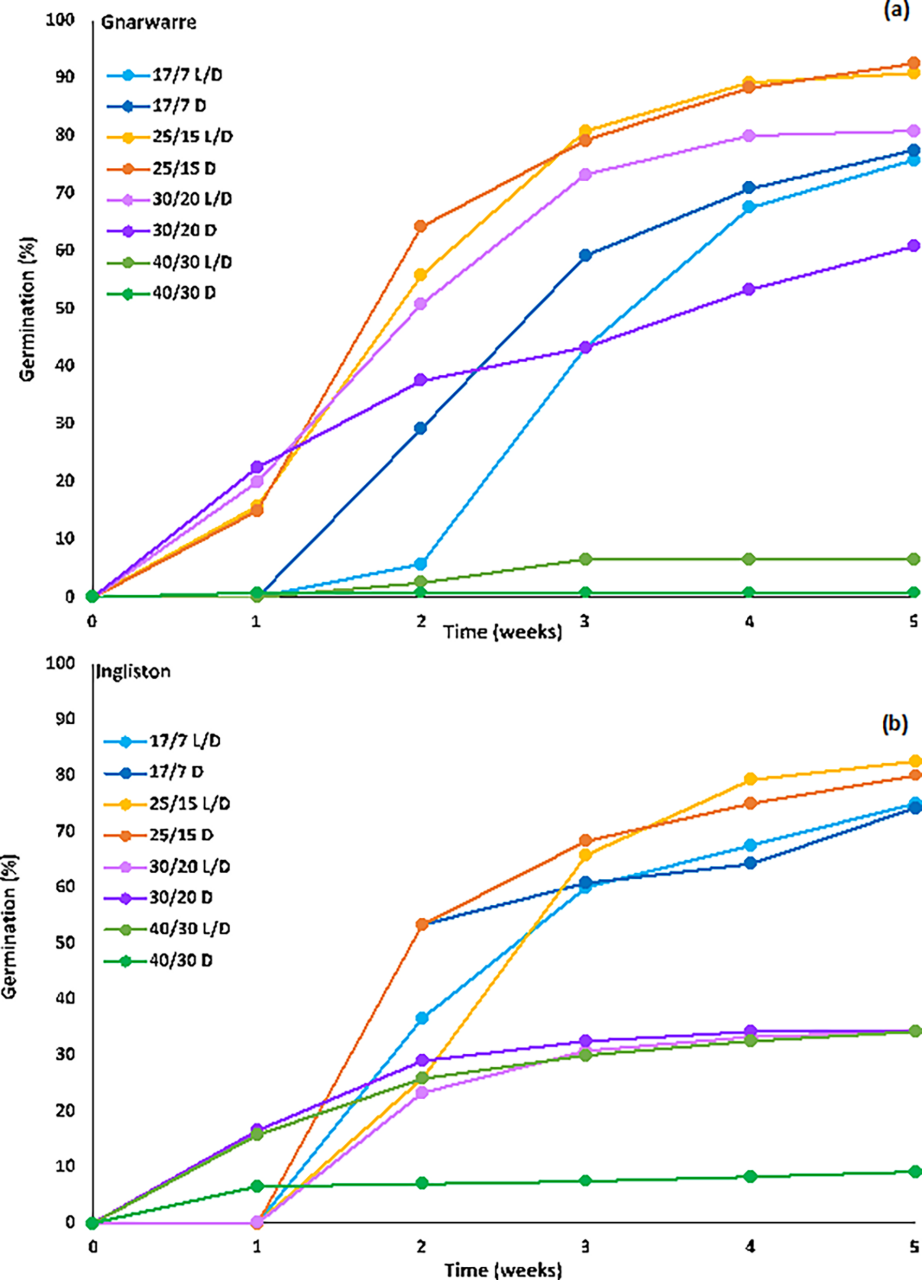
**a The germination (%) for the Ingliston and b: Gnarwarre populations of *Nassella trichotoma* seeds tallied at the end of every week from day 0 for the alternating temperature and photoperiod regime treatment**.

Germination rate was steady, with both populations taking two weeks before 50% germination or higher was observed (Fig 3A and 3B). For the Ingliston population, 53.3% germination was observed for both the 17/7°C and 25/15°C temperature regimes under complete darkness after two weeks of incubation, which was higher than the germination (%) observed for the alternating photoperiod at the same temperature, being only 36.6% and 25.8%, respectively. A similar result was also observed in the Gnarwarre population for the 25/15°C temperature regime under both photoperiod treatments, with 55.8% and 64.2% germination being observed for the alternating and complete darkness photoperiods respectively. Unlike the Ingliston population, Gnarwarre had a lower germination (%) at the 17/7°C temperature treatment, with only 29.2% germination observed under alternating and 5.8% germination in complete darkness after two weeks of incubation. However, at the two-week mark, the Gnarwarre population had 50.8% germination in the 30/20°C alternating photoperiod treatment, which was much higher than the 23.3% germination observed in the Ingliston population at the same point in time. These results show that for the Ingliston population, the temperatures of 17/7°C and 25/15°C under complete darkness favours more rapid germination rates, while the Gnarwarre population demonstrated a faster germination rate under the temperature regimes of 25/15°C and 30/20°C, with light being an irrelevant factor. After three-weeks of incubation, the germination (%) rate declined for all the tested treatments.

For many plants, light plays an important role in allowing a seed to gauge its position within the soil profile, identify existing competition, and detect any soil disturbance events [1]. For *N*. *trichotoma*, light was not observed to be an important factor for regulating germination when alternating temperatures were favourable. Photoperiod as a singular factor was only significant in the 30/20°C alternating temperature for the Gnarwarre population with only 60% of the seeds germinating compared with 80% in the alternating light and dark trials. Germination in complete darkness indicates that *N*. *trichotoma* is non-photoblastic; rather, other environmental factors may be more closely linked with breaking its dormancy [1, 16, 52]. The germinated seedlings from the complete darkness treatment exhibited etiolated growth, while those seedlings from the alternating photoperiod treatment were observed to be larger and a vibrant green colour. Light also had little influence on the rate of germination, with both tested photoperiods producing similar weekly germination yields. Germination was highest in both populations at the alternating temperatures of 17/7°C (approximately 75% for Ingliston and 76% for Gnarwarre) and 25/15°C (81% for Ingliston and 91% for Gnarwarre). The *in situ* average maximum temperature of the two populations is approximately 25°C and 15°C, respectively, in the spring and summer months, and 15°C and 5°C, respectively, throughout winter, which is fitting with this optimal temperature result [46].

There was a significant difference between the two populations when exposed to the higher two alternating temperature regimes. The Ingliston population experience significantly higher germination at the 40/30°C regime than that of the Gnarwarre population. In the 30/20°C and the 40/30°C alternating photoperiod treatments, germination was reduced to a similar level for the Ingliston population, indicating that if moisture levels are adequate, approximately 34% of this population’s seeds will still germinate at these unfavourable temperatures. The Gnarwarre population experienced an exponential reduction in the 40/30°C treatment, with only 6% of the seeds germinating under alternating light and dark conditions, and no seeds germinating under complete darkness at this temperature. The Gnarwarre population had significantly higher germination in the 30/20°C treatment (80% in alternating light and 60% in complete darkness) compared to the Ingliston population. The average seasonal temperatures are slightly warmer across all seasons at Gnarwarre compared to Ingliston, which may have contributed to this population’s higher optimal germination temperatures [11]. These results observed subtle variations in germination response to alternating temperature regimes, and it is possible that these differences could be stronger between more spatially distinct populations.

### Effect of drought

**Fig 4:**
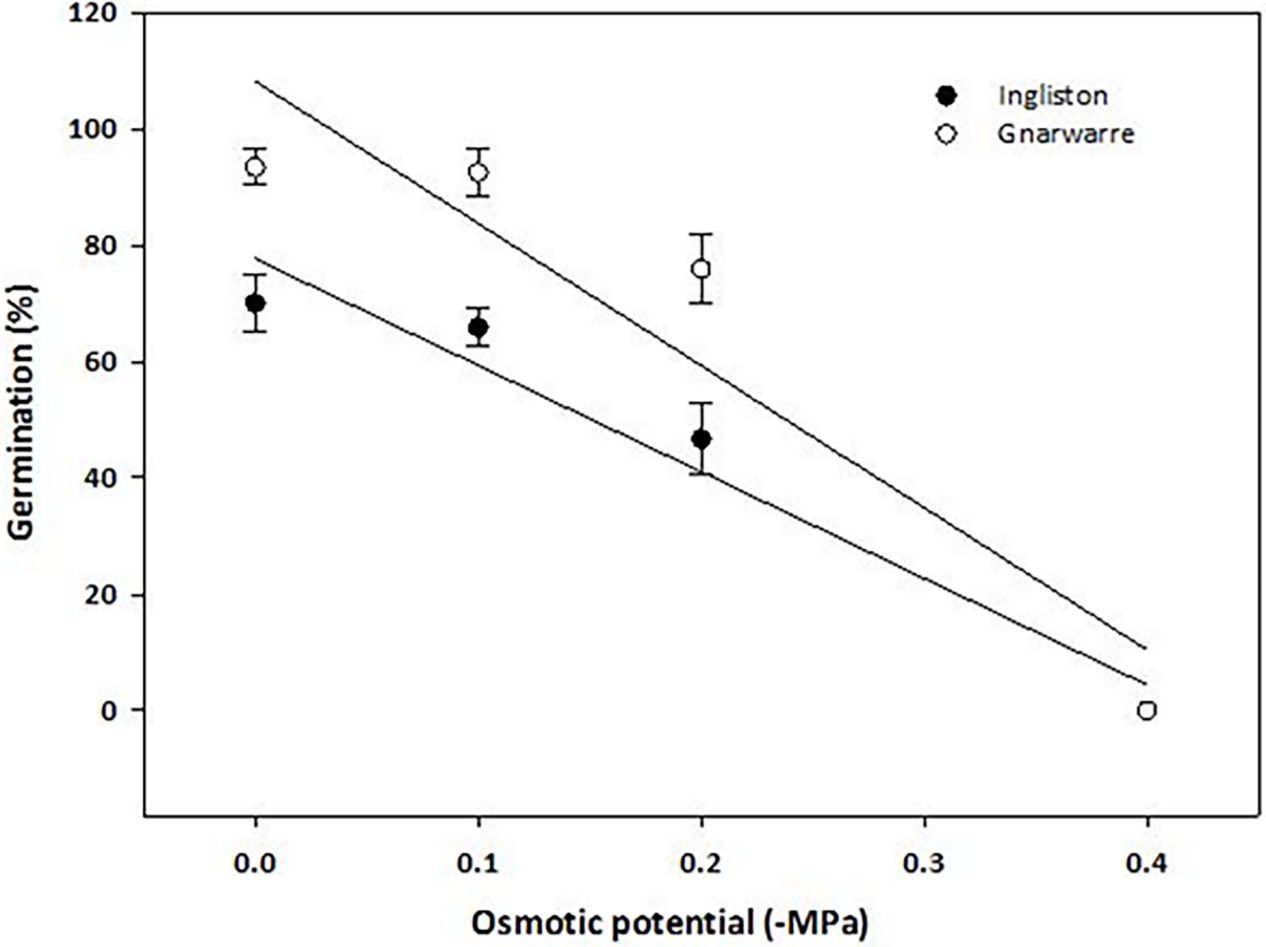
The effect of osmotic potential (-MPa) on the germination (%) of *Nassella trichotoma* for Ingliston (white dot) and Gnarwarre (black dot) after incubation in a growth chamber at 25/15°C 12 hours light/12 hours dark for 30 days. The line for Ingliston represents a linear polynomial model fitted to the data with the equation G% = 77.671+83.09*X. The osmotic potential for 50% inhibition of maximum germination for Ingliston is estimated as -0.15MPa. The same model was fitted to the Gnarwarre data with the equation G% = 108.67+24.43*X. The osmotic potential for 50% inhibition of maximum germination for Gnarwarre is estimated as -0.24MPa. The vertical bars represent standard error of the mean.

For the drought treatment, germination was highest in the control for both populations with the Ingliston population having 70% germination and Gnarwarre having 93.3% germination (Fig 4). There was little variation in germination (%) for the osmotic potential of 0.1MPa with Ingliston having 65.8% germination and Gnarwarre having 92.5% germination. Exposure of the seeds to an osmotic potential of 0.2MPa resulted in a decline in germination within each population, as compared to 0.1MPa, with the germination (%) in the Ingliston population declining to 46.6% and 75.8% for the Gnarwarre population. Both populations had significantly reduced germination at the osmotic potential of 0.4MPa and above, with zero germination being observed from this concentration onwards (p = 0.000). The Gnarwarre population had significantly higher germination compared to the Ingliston population in the control, 0.1MPa and 0.2Mpa treatments (p = 0.000), suggesting that the Gnarwarre population was able to germinate better under the effect of drought than the Ingliston population (p = 0.000) (Table 1). The r-squared value of 96% demonstrates that the effect of this osmotic potential treatment strongly inhibited *N*. *trichotoma’s* seed germination at concentrations of 0.4MPa and above.

### Effect of salinity

**Fig 5:**
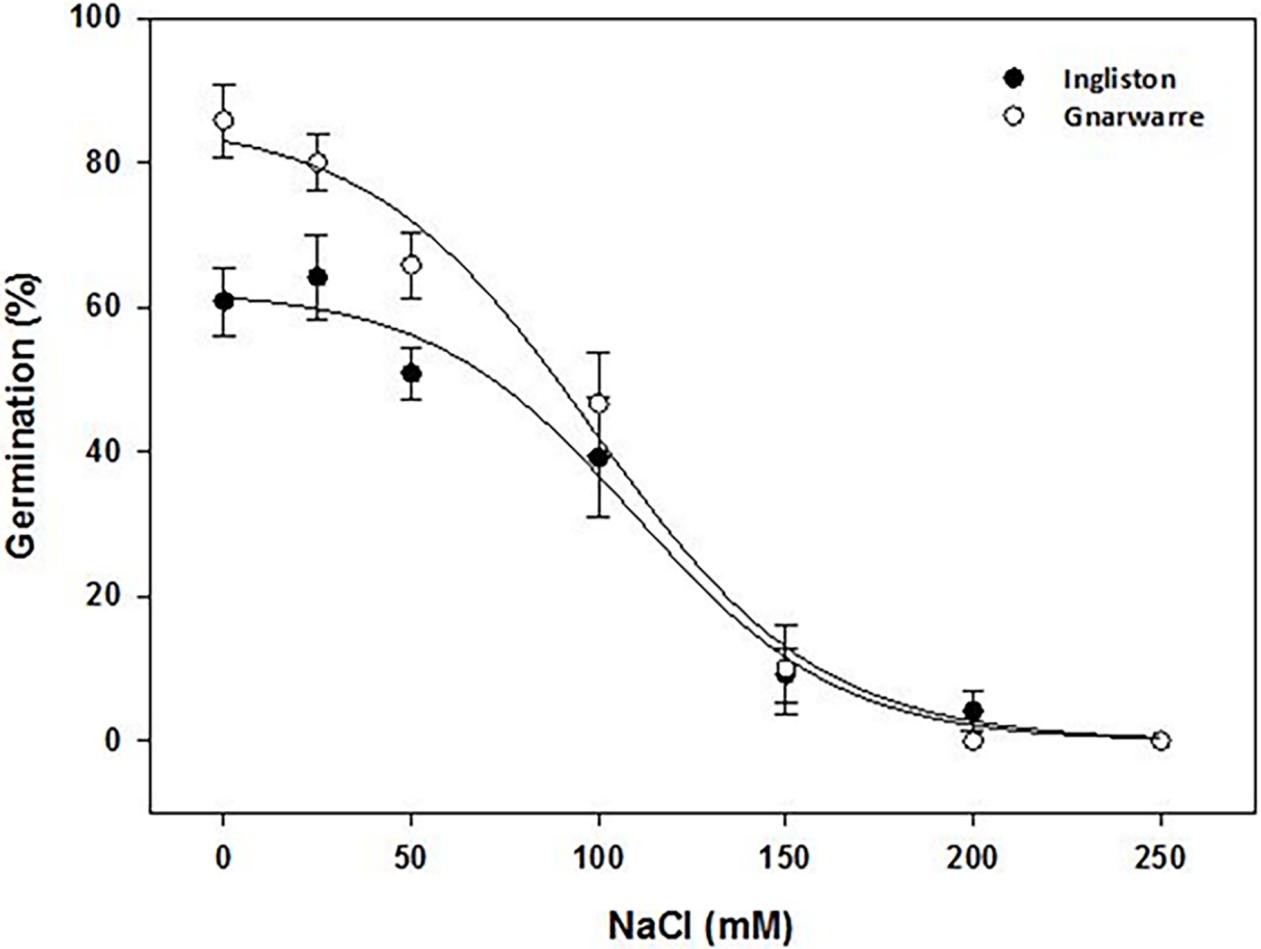
The effect of NaCl (mM) on the average germination (%) of *Nassella trichotoma* for Ingliston (black dot) and Gnarwarre (white dot) after incubation at 25/15°C 12 hours light/12 hours dark for 30 days. The Ingliston population was fitted with a three-parameter sigmoid model with the equation G% = 62.45/(1+e(-x-109.6/27.22). The Gnarwarre population was also fitted with a three-parameter sigmoid model with the equation G% = 86.02/(1+e(-x-98.6/29.79). Germination was reduced to 50% at a NaCl concentration of 109.6mM for the Ingliston population and 98.6mM for the Gnarwarre population. The vertical bars represent standard error of the mean.

For the salinity treatment, the highest germination (%) for the Ingliston population of 64.1% was obtained in the 25mM treatment, and the highest germination (%) for the Gnarwarre population of 85% was obtained in the control treatment (Fig 5). Significantly higher germination in the Gnarwarre population compared to the Ingliston population in the control and 25mM treatments (p = 0.001), and this was independent of the salinity treatment (p = 0.031) (Table 1). A NaCl concentration of 150mM significantly reduced *N*. *trichotoma’s* germination in both populations (p = 0.000), with only 9.1% germination being observed for the Ingliston population and 10% for the Gnarwarre. The model suggests that the Gnarwarre population could tolerate up to 71.63mM of the NaCl solution before germination was inhibited by 50%, while the Ingliston population germination was reduced to 50% with a NaCl concentration of 55.99mM. Germination continued to decline as the concentration of NaCl increased, and zero germination was observed in both populations in the 250mM treatment. The r-squared value of 89% confirms that the salinity treatment was the main factor reducing seed germination (%).

Drought and salinity are environmental factors that impose osmotic stress on seeds, preventing the natural flow of water into the seed from its surrounding environment. Under osmotically stressful conditions, seeds may be unable to achieve the critical moisture levels required for imbibition, and therefore unable to prepare for germination. The results of this study demonstrated that water availability was a highly influential factor for triggering *N*. *trichotoma* seed germination. In the drought treatments, both populations demonstrated reasonable germination rates under the osmotic stress of -0.2MPa (46.6% for Ingliston and 75.8% for Gnarwarre), but doubling this stress to -0.4MPa completely inhibited germination in both populations. The effect of osmotic stress had a similar effect on *C*. *nutans*, where germination was observed to be somewhat unaffected by osmotic stress between 0 and 0.2MPa, but was almost completely inhibited by -0.4MPa [22]. A tetrazolium test identified a high proportion of the un-germinated *N*. *trichotoma* seeds were still viable at the conclusion of the trials, indicating that these seeds may germinate if osmotic conditions became favourable [52].

The Gnarwarre population had a higher tolerance to osmotic stress than the Ingliston population. At an osmotic potential of -0.2MPa, Gnarwarre had significantly higher germination than that of the Ingliston population. The home ranges of the two populations have substantial variations in the volume of rainfall, with Ingliston having an average yearly rainfall of 654mm, while the average for Gnarwarre is only 437mm, in addition to this the pattern of rainfall for Ingliston is higher than Gnarwarre across all seasons, particularly in the autumn months which is when *N*. *trichotoma’s* non-deep dormancy begins to break [36, 46, 53]. In addition to this, the average maximum temperature is lower at Ingliston compared to Gnarwarre, meaning this site is likely to have higher soil moisture conditions and exert less osmotic stress on seeds and mature seed producing plants. The Gnarwarre population is subjected to lower rainfall and warmer maximum temperatures, therefore the osmotic pressures of this environment is selecting for those plants that are more tolerant of dryer conditions compared to Ingliston. The results suggest that the different osmotic selective pressures of these two environments have resulted in variations in the seeds sensitivity to osmotic stress.

Salinity exerts a similar osmotic stress as drought, however as a result of the increased ion concentrations, saline conditions can have a more profound inhibiting effect on seed germination [4, 54, 55]. While salinity often has a dormancy inducing effect on seeds, some salt tolerant species, like *Vicia faba* [56], *Atriplex lentiformis* [56] and *Juncas ranarius* [57] have been observed to germinate under higher salinity stress, however the rate and vigour of germination is considerably reduced. In addition to this, salinity can reduce a seedlings ability to take up nutrients, such as potassium ions, and accumulate higher proportions of sodium and chloride ions, reducing the seedlings growth potential [54, 58]. The germination inhibiting effect of increasing salinity concentrations was similar in both populations. The Gnarwarre population proved to have greater germination (%) than the Ingliston population, particularly in the control and 25mM treatments. The 100mM treatment reduced germination to 47% in the Gnarwarre population and to 39% in the Ingliston population, which was significantly lower than the control treatments. Germination was reduced to 9.2% and 10% for Ingliston and Gnarwarre respectively in the 150mM treatment, and the 200mM treatment reduced germination of the Ingliston population to only 4.2%, and no germination occurred for the Gnarwarre population at this concentration. No further germination occurred beyond 150mM indicating that high salinity concentrations have an inhibiting effect on the germination volume of *N*. *trichotoma* seeds. The Gnarwarre collection site had moderately soil salinity (4.3dS m-1) compared the Ingliston site’s soil being only slightly saline soil (3.8dS m-1), therefore the greater germination observed in the Gnarwarre population could be attributed to this environmental selective pressure. Similar responses to salinity have been observed in other noxious weeds, including *Amaranthus spinosus* [28], *Croton setigerus* [22], and *Emex australis* [52]. The inhibiting effect of salinity on seed germination can explain why mature *N*. *trichotoma* plants are rarely seen growing in saline affected regions of Australia, and the small proportion of the population that do germinate in these regions are outcompeted with more salt tolerant plants [14, 19, 36, 53].

### Effect of burial depth on seedling emergence

**Fig 6:**
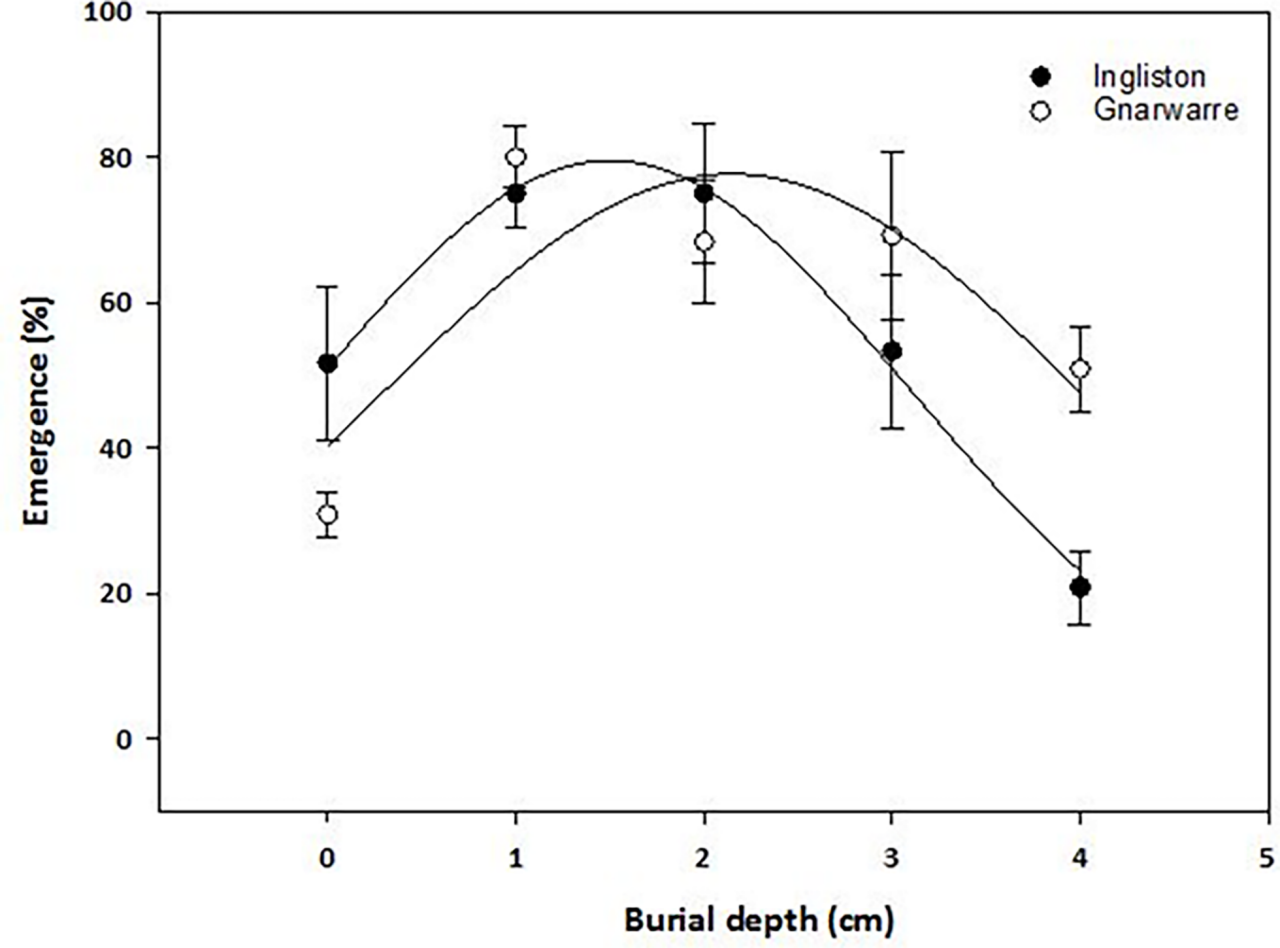
The effect of seed burial (cm) on the average seedling emergence (%) of *Nassella trichotoma* for Ingliston (black dot) and Gnarwarre (white dot) after incubation at 25/15°C 12 hours light/12 hours dark for 30 days. The Ingliston population was fitted with a three-parameter peak Gaussian model with the equation E(%) = 79.53*e(-0.5*X-1.49/1.59)^2^. The Gnarwarre population was also fitted with a three-parameter sigmoid model with the equation E(%) = 77.68*e(-0.5*X-2.15/1.88)^2^. Maximum emergence occurred at a burial of 1.49cm for Ingliston and 2.15cm for Gnarwarre. The vertical bars represent standard error of the mean.

The burial depth treatment obtained the highest emergence (%)at the 1cm burial depth treatment for both populations, with the Ingliston population having 75% seedling emergence and the Gnarwarre population having 80% emergence (Fig 6). The Ingliston population had the same proportion of seedlings emerge at the 2cm burial treatment. A variation was observed between the two populations in the surface treatment and the 4cm burial treatment (p = 0.045). In the surface treatments, the Ingliston population had an emergence (%) of 51.6% compared to only 30.8% for the Gnarwarre population. Contrastingly, in the 4cm burial treatment, Gnarwarre had 50.8% emergence, while Ingliston had only 20.8%. Despite an identifiable bell-curve response to the effect of seed burial in both populations, the r-squared value of only 49% suggests that other factors may have also been influencing the results of this treatment.

### Effect of burial depth on seed germination and viability under field conditions

**Fig 7:**
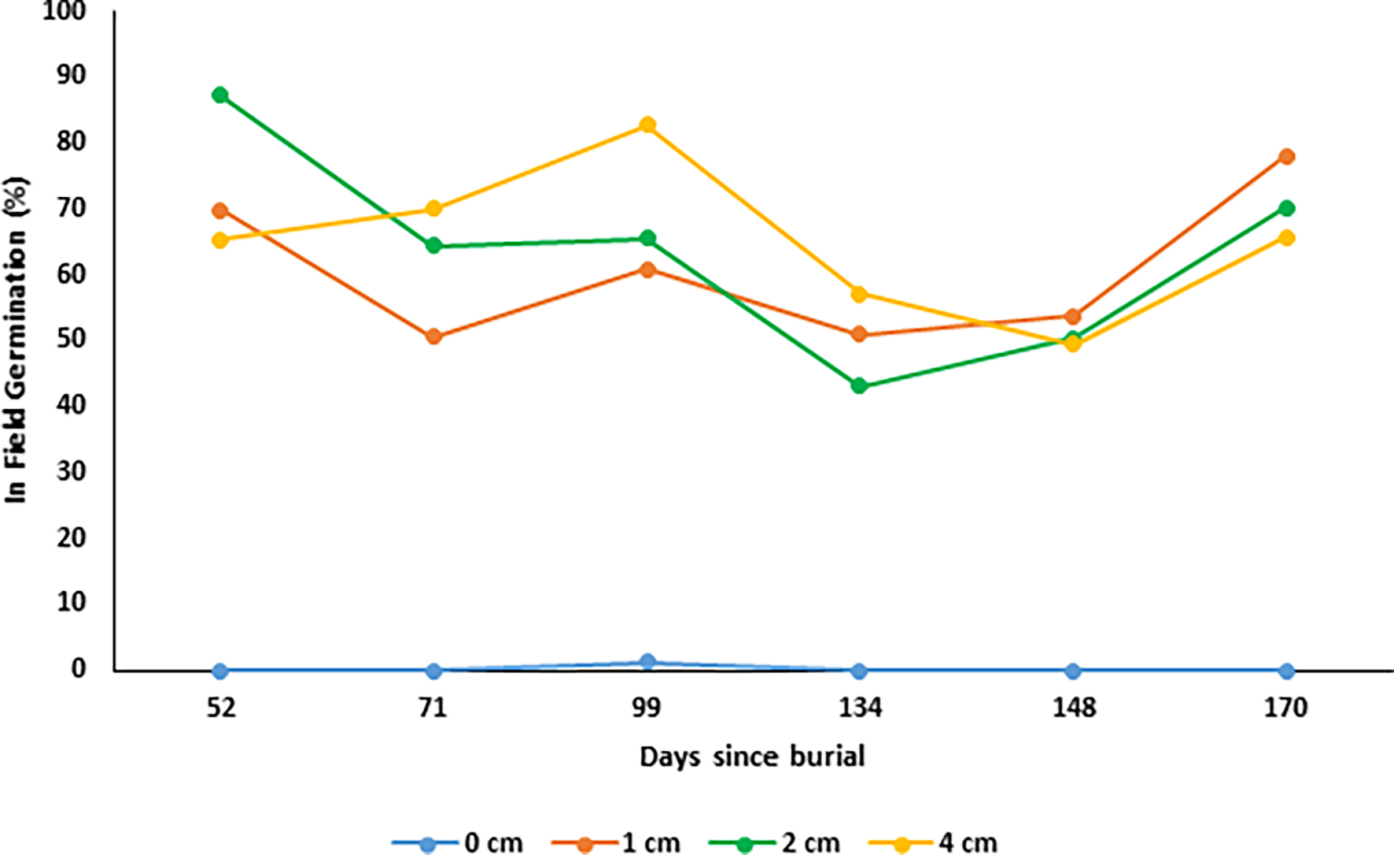
The effect of seed burial (cm) under field conditions on seed germination (%) for *Nassella trichotoma*. Each month 120 seeds were collected from each depth and this graph shows the proportion (%) of seeds that had germinated within the field.

**Fig 8:**
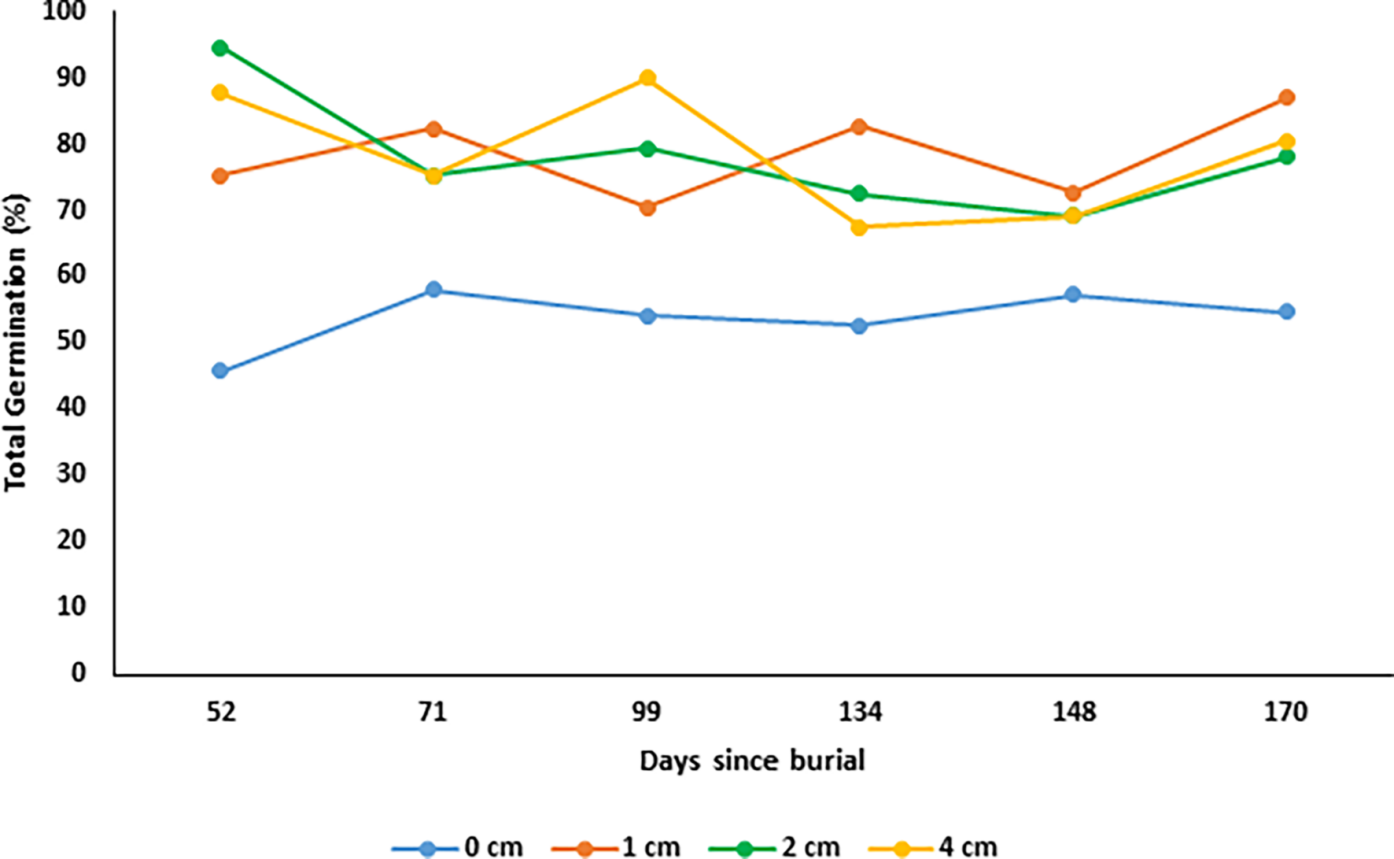
The effect of seed burial (cm) under field conditions on total seed viability (%) for *Nassella trichotoma* collected from Ingliston. Each month 120 seeds were collected from each depth and then incubated for up to 30 days and then had a TTC viability test conducted on the seeds. This graph shows the total number (%) of seeds that had germinated within the field, within the incubation period, and responded positively to the viability test.

The results of the seed germination under field conditions treatment, shows that *N*. *trichotoma* demonstrated similar germination (%) at 1, 2 and 4cm burial depth under field conditions (Fig 7). The germination (%) observed at these depths were significantly higher than the germination on the soil surface (0cm) (p = 0.000). The seeds viability remained consistent throughout the six-month collection span, which suggests that seeds have the ability to remain viable under field conditions for at least 170 days (Fig 8). Burial of 1cm or deeper appeared to have a protective effect on the seeds, as these seeds had higher viability compared to those exposed to surface conditions.

The effect of burial depth influenced the emergence (%) of seedlings slightly differently between the two populations. The depth of 1cm was optimal for seedling emergence with 75% and 80% germination for Ingliston and Gnarwarre respectively, with the Ingliston population also having the same proportion of seeds germinate at a burial depth of 2cm. A burial depth of 4cm had significantly different proportion of emergence between the two populations, with Gnarwarre having 50% emergence at this depth, while Ingliston’s emergence was reduced to only 20%. As it was identified in the photoperiod treatment, *N*. *trichotoma* does not require light for germination, therefore it is likely that the significant difference is related to seedling vigour. Lighter seed weight was observed to increase sensitivity to burial depth interspecifically amongst 13 different annual species collected from a Spanish grassland [59]. Intraspecific variations in the seed size was observed in two spatially distinct populations of *Caucalis platycarpos* [38] and *Ambrosia artemisiifolia* [60] as a response to different environmental pressures, allowing the population with larger, denser seeds to have higher emergence from greater burial depths [59]. A similar variation in the seed density was observed between the two *N*. *trichotoma* populations studied, which may have influenced the difference in emergence at the 4cm depth. The average individual seed weight of the Gnarwarre seeds were heavier (0.86mg) than the Ingliston seeds (0.76mg), which could explain the significant difference in emergence at this depth. It was identified in the artificial aging under field conditions treatment that germination of more than 20% does occur at a burial of 4cm in the Ingliston population, in fact, an average of 65% of the seeds germinated at this depth. Therefore, the lighter density of the Ingliston population seeds is the likely factor decreasing emergence.

*Nassella trichotoma* seeds experienced a significant reduction in germination and seedling emergence at the surface treatments compared to 1cm burial in both populations, with Ingliston being reduced to 50% and Gnarwarre to 30% germination. Under field conditions, only one seed germinated on the soil surface across the 6 months tested, and the total viability results indicated that surface conditions reduce seed viability compared to a burial of 1cm or greater. This is somewhat uncommon in species that germinate well in alternating light and dark regimes, as surface conditions have been identified to be favourable for optimal germination in a magnitude of weed species inclusive of: *Chromolaena odorata* [61], *Ceratocarpus arenarius* [62], *Galinsoga quadriradiata* and *Galinsoga parviflora* [8]. Germination and emergence of the noxious grass weed *E*. *colona*, was significantly reduced from 97% at the soil surface to 12% with a burial depth of only 0.5cm [5]. A possible reason for the reduced germination in the surface burial treatment may be related to a defensive response of the seeds far-red phytochromes, as these play an important role in identifying the optimal time for germination by not only sensing the intensity of competition, but also excessive light associated with soil surface conditions [12]. This mechanism is known as high irradiance response sensitivity and it protects the seed from germinating under intense sunlight as these factors can indicate harsh and unfavourable temperatures and dry conditions [12]. As it was identified in the photoperiod trials, *N*. *trichotoma* is non-photoblastic and can germinate well with alternating light and dark conditions and in complete darkness, furthermore the results of the drought treatment highlighted that *N*. *trichotoma* germination is highly dependent on ample water availability. The yearly average solar exposure for Ingliston and Gnarwarre is 15.1 MJ/m^2^ and 15.2 MJ/m^2^ respectively, which indicates that these sites experience predominantly overcast conditions, and tolerating full sunlight would not be a selective pressure of these environments [46]. Throughout the burial depth experiment, it was observed that the surface conditions experienced loss of soil moisture quicker that the soil layers just below, despite regular watering. Therefore, it is likely that in addition to the far-red phytochromes preventing germination under full sunlight, difference in soil moisture between the surface and 1cm burial treatments also may have influenced the significant difference observed in germination and emergence at these depths.

### Effect of exposure to radiant heat under increasing time durations

**Fig 9:**
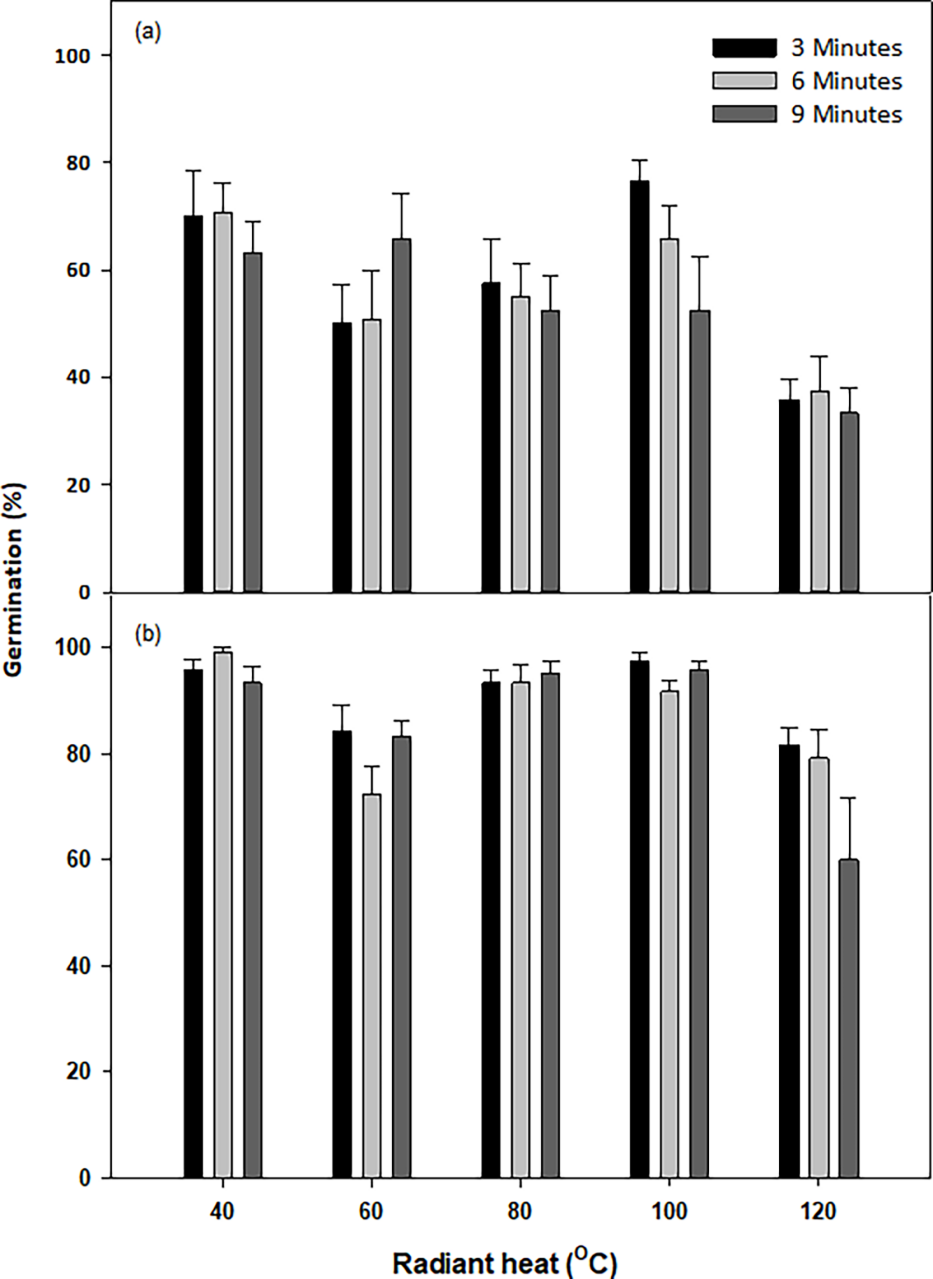
**a: The effect of exposing *Nassella trichotoma* seeds to radiant heat (**^**O**^**C) at increasing time durations (minutes) on germination (%) of for the Ingliston, b: Gnarwarre populations after incubation in a growth chamber at an alternating temperature of 25/15**^**°**^**C 12 hours light/12 hours dark for 30 days.** Vertical bars represent standard error of the mean.

Pre-exposure to radiant heat had a somewhat positive influence on germination (%) of both *N*. *trichotoma* populations (Fig 9A and 9B). Exposure to the 120°C treatment reduced Ingliston’s, germination (%) to 35.8%, 37.5% and 33.3% for the 3, 6 and 9 minutes durations, respectively. This reduction was significantly lower than the 40°C treatments for this population (p = 0.000). None of the temperatures or exposure durations resulted in germination (%) of less than 50% for Gnarwarre, and this population experienced significantly higher germination than the Ingliston population at all tested treatments (p = 0.000). The lowest germination for the Gnarwarre population of 60% was obtained when seeds were exposed to 120°C for 9 minutes.

The Gnarwarre population responded positively to radiant heat, particularly in the 40, 80 and 100°C treatments, which produced higher germination (%) than the optimal photoperiod and temperature treatment. For this population, germination was only reduced to 60% when exposed to 120°C for nine minutes. The Ingliston population showed greater sensitivity to radiant heat, with no heat treatments producing better germination than the optimal photoperiod and temperature regimes. Germination was reduced to approximately 50% in the 60 and 80°C treatments, and to only 35% in the 120°C treatments. Despite this, germination proportions between 76 and 50% in newly burnt areas could still give the Ingliston population a decent competitive advantage. There was no significant difference to germination for either population as a result of the duration of heat exposure. These results highlight the importance for integrating fire management with weed management, as fire has been observed to enhance weed invasion, particularly in areas with poor nutrient availability such as roadsides [63]. Fire was observed to enhance the rate and volume of germination in the invasive pastoral grass *Hyparrhenia rufa*, despite it killing most of the established population [64]. The seeds of *N*. *trichotoma* are fire tolerant to temperatures of at least 120°C and germination and seedling recruitment is enhanced by heat. Furthermore, the reduced competition associated with burning will likely promote the recruitment of this opportunistic weed.

### Effect of pH on germination and variations in germination

**Fig 10:**
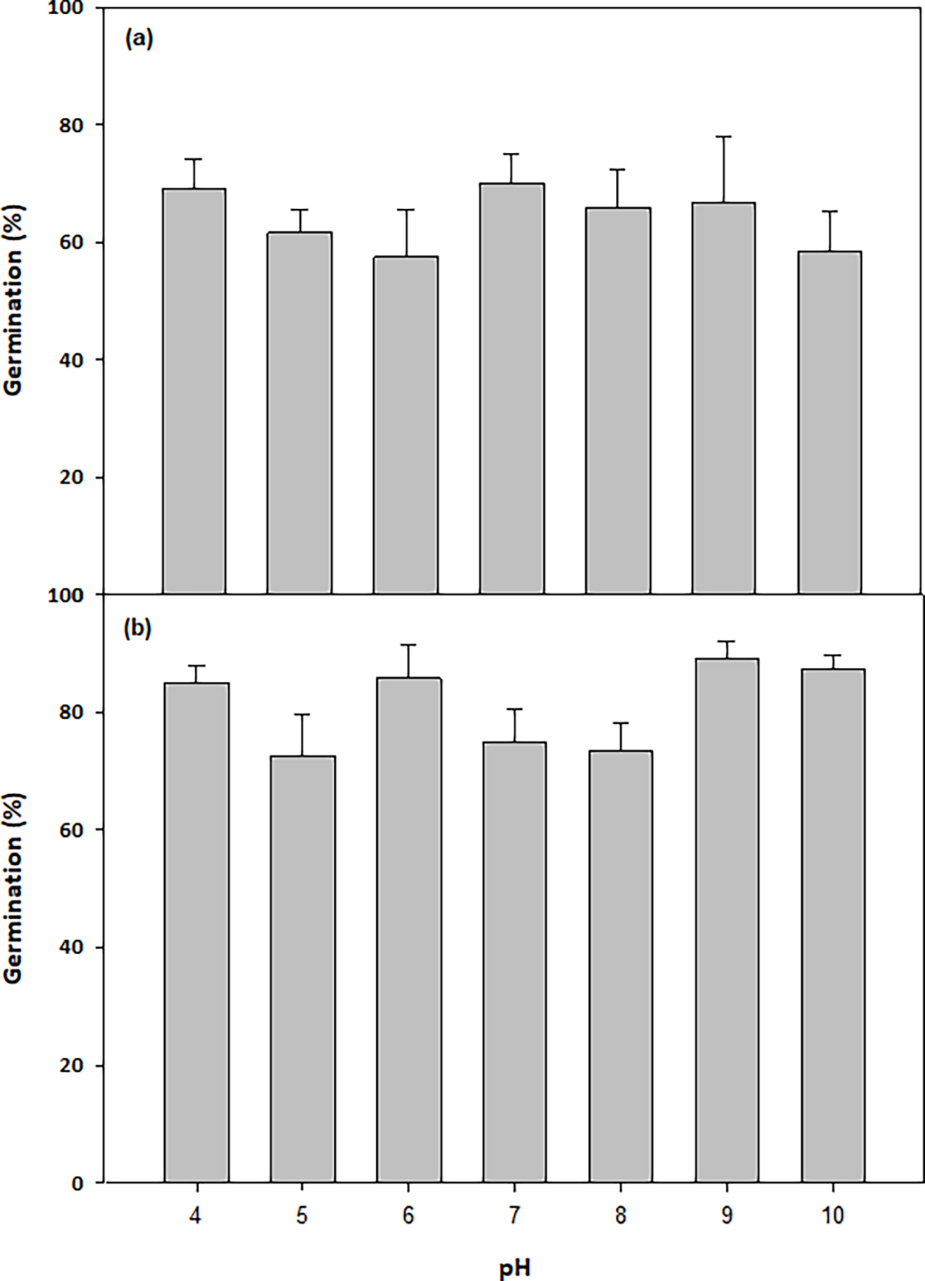
**a: The effect of pH on the germination (%) of *Nassella trichotoma* seeds collected from Ingliston and Gnarwarre, b: after incubation in a growth chamber at an alternating temperature of 25/15**^**°**^**C 12 hours light/12 hours dark for 30 days.** Vertical bars represent standard error of the mean.

Tthe range of pH levels tested did not have a significant effect on the germination (%) within either population (Fig 10A and 10B). The germination (%) was significantly higher in the Gnarwarre population compared to the Ingliston population across all the tested treatments (p = 0.000), however this was not linked to the pH level (p = 0.244). The r-squared value of 36.6% suggests that the pH treatment was not the dominant factor influencing these differences. Despite this variation, both populations responded with a similar trend to the range of pH levels treated.

This study highlighted that *N*. *trichotoma* does not have a significant preference for a particular pH level, and both populations were able to germinate well across the tested range of pH 4 to 10. Despite both populations being collected from sites with acidic soils, the lower pH levels tested were not favoured any more than the higher levels, suggesting that soil pH is not an active selective pressure on either population. The generalist attributes of most weeds allows them to take advantage of a wide range of soil types as this allows them to exploit a magnitude of environments, including disturbed and degraded regions. The minor effect of pH levels on successful weed seed germination has also been observed in *Amaranthus retroflexus* [28], *Galenia pubescens* [34] and *Nicotiana glauca* [30]. The Gnarwarre population had higher germination than the Ingliston populations at all pH levels, however the r-squared value of 36.6% indicates that this is unlikely to be a result of the pH treatment. Overall, the Gnarwarre population had higher seed viability than the Ingliston population. The variation observed in weight could account for the difference in the total proportion of germination. The Gnarwarre seeds were heavier and denser than the seeds collected from Ingliston, and greater seed density has been observed to promote higher germination yields [59].

## Conclusion

The results of this study highlight that *N*. *trichotoma* seeds are non-photoblastic, and dormancy break can be triggered by favourable of alternating temperatures of approximately 25/15°C and ample water availability. Radiant heat was also observed to have a positive effect on total germination yields. Under osmotic stress and salinity, germination was significantly reduced, and water appeared to be the most important limiting factor on germination. Seeds are able to germinate when buried to a depth of at least 4cm, and seedling emergence can occur at this depth, although the success of emergence appears to be linked to seed weight, with the denser Gnarwarre seeds having higher emergence than the lighter Ingliston seeds at this depth. Germination was not enhanced or inhibited by pH level, suggesting that soil pH is not a limiting factor on this species recruitment.

These findings suggest that light reducing management techniques will be unsuccessful for preventing germination. Tilling the seeds to a depth of at least 4cm may reduce the emergence of seedlings, and because the seeds still germinate when buried, this may quickly reduce the seedbank. The effect of seed burial on emergence should be further explored by investigating the effect of greater seed burial depths under controlled and field conditions so that better recommendations can be made for using tillage as a control method. Land managers should look for *N*. *trichotoma* recruitment after good rainfall events and suitable temperature regimes, particularly after fire treatments. By understanding the climatic conditions that significantly enhance recruitment, management techniques can be applied accordingly to maximise their productivity.

This study observed variations in the seed ecology between the two populations of *N*. *trichotoma*, and it is likely that greater variations would be observed between populations with greater differences in selective pressures. It would be beneficial to observe the spatial variations between populations across different states of Australia, or even internationally in order to develop a more thorough understanding of this species seed ecology so that management recommendations can be made confidently across wide geographical gradients.

## Supporting information

S1 File (XLSX)
